# Evaluation of CK8/18 and CK19 Expression as Adjunct Immunohistochemical Markers in Non-Keratinizing Nasopharyngeal Carcinoma

**DOI:** 10.3390/diagnostics15182292

**Published:** 2025-09-10

**Authors:** Ummul Afila Omar, Suria Hayati Binti Md Pauzi, Mohd Razif Bin Mohamad Yunus, Rosnah Sutan, Nur Maya Sabrina binti Tizen Laim, Muaatamarulain bin Mustangin, Reena Rahayu Md Zin

**Affiliations:** 1 Department of Pathology, Universiti Kebangsaan Malaysia, Kuala Lumpur 56000, Malaysia; 2 Department of Otorhinolaryngology, Head & Neck Surgery, Universiti Kebangsaan Malaysia, Kuala Lumpur 56000, Malaysia; 3 Department of Public Health Medicine, Universiti Kebangsaan Malaysia, Kuala Lumpur 56000, Malaysia; 4 Department of Pathology, Faculty of Medicine, Universiti Kebangsaan Malaysia, Kuala Lumpur 56000, Malaysia

**Keywords:** CK8/18, CK19, nasopharyngeal carcinoma

## Abstract

**Background:** Nasopharyngeal carcinoma (NPC), particularly the non-keratinizing subtype (NK-NPC), is prevalent in Southeast Asia and often presents diagnostic challenges due to overlapping histological features with benign nasopharyngeal lesions. While Epstein–Barr virus (EBV) serology supports diagnosis in many cases, its limitations in sensitivity and specificity necessitate additional tissue-based markers. **Objective:** To assess the immunohistochemical expression of cytokeratins CK8/18 and CK19 in NPC compared to benign nasal tissue and evaluate their potential as adjunct immunohistochemical markers. **Methods:** This retrospective study evaluated the immunohistochemical expression of cytokeratins CK8/18 and CK19 in 24 NK-NPC and 22 benign nasopharyngeal tissue samples collected between April 2021 and April 2024 at Hospital Canselor Tuanku Muhriz, Kuala Lumpur, Malaysia. Staining intensity and distribution were scored semi-quantitatively, and statistical analysis was performed using SPSS v29.0 (*p* < 0.05). Results: CK19 was expressed in all NK-NPC cases, with strong positivity in 79.2%, while CK8/18 was positive in 92%, primarily with weak to moderate staining. Only one benign case (inverted papilloma) showed focal positivity. The differences in expression between malignant and benign tissues were statistically significant (*p* < 0.001). Sub-analysis of EBER-positive cases (*n* = 15) confirmed consistently strong CK19 expression. **Conclusions**: Based on this small retrospective cohort, CK8/18 and particularly CK19 demonstrate expression patterns that may support their use as adjunct immunohistochemical markers in the histopathological assessment of NK-NPC, especially in morphologically ambiguous cases. Further validation in larger studies is needed before these markers can be considered for routine diagnostic application.

## 1. Introduction

Nasopharyngeal carcinoma (NPC) is a malignant epithelial neoplasm of the nasopharynx that typically exhibits squamous differentiation. The most common site of origin is the lateral wall of the nasopharynx, particularly the Fossa of Rosenmüller, followed by the superior posterior wall. Globally, NPC is rare in most regions, with age-standardized incidence rates (ASR) of less than 1–2 per 100,000 as of 2020. However, its incidence is markedly higher in specific regions such as China and Southeast Asia [1]. In Malaysia, NPC ranks among the five most common cancers and is the fifth most common cancer in men, with an ASR of 6.4 per 100,000 males from 2012 to 2016 [2]. The highest incidence is observed among the Chinese population, followed by Malays and Indians. A study conducted in Sarawak between 1996 and 1998 reported that the Bidayuh ethnic group had the highest documented NPC rate globally, at 31.5 per 100,000 males [3]. Alarmingly, nearly 40% of Malaysian cases are only diagnosed at stage IV, when the five-year survival rate declines significantly from 98% in stage I to 73% [2,4].

According to the latest World Health Organization (WHO) classification, NPC is categorized into three histological subtypes: non-keratinizing squamous cell carcinoma (NK-NPC), keratinizing squamous cell carcinoma (K-NPC), and basaloid squamous cell carcinoma [1]. NK-NPC is the most common subtype and is strongly associated with Epstein–Barr virus (EBV) infection. This association underpins the use of EBV serology as a screening tool, typically involving serum IgA antibodies against viral capsid antigen and early antigens. However, the sensitivity and specificity of EBV serology are limited: not all seropositive individuals develop NPC, and not all NPC cases particularly keratinizing subtypes are EBV-related [5]. This inconsistency has prompted the search for more reliable and universally applicable biomarkers for NPC screening and diagnosis.

Cytokeratins (CKs), a family of intracytoplasmic intermediate filament proteins found in epithelial cells, have garnered interest in cancer diagnostics and prognostics. CKs are divided into two types based on molecular weight and isoelectric point: type I (acidic, low molecular weight, CK9–CK20), encoded on chromosome 17q; and type II (basic/neutral, high molecular weight, CK1–CK8), encoded on chromosome 12q [6]. CK8 and CK18 are typically co-expressed and function as a structural pair within gland-forming epithelial cells [7]. While CK18 is largely absent in normal squamous epithelia, it is consistently expressed in various adenocarcinomas, including those of the liver, kidney, and gastrointestinal tract and has also been proposed as a prognostic marker in breast and colorectal cancers [8,9,10]. In squamous cell carcinomas, CK18 expression is frequently upregulated in high-grade or metastatic lesions, such as oral and esophageal squamous cell carcinomas, and is associated with aggressive behavior [11,12].

Similarly, CK19—a type I acidic keratin—is expressed in a variety of glandular epithelial tissues including the gastrointestinal tract, bile ducts, respiratory epithelium, and salivary glands [6] In malignancy, CK19 expression is retained and widely used as a diagnostic marker, for example in thyroid carcinoma [13] and in distinguishing hepatocellular carcinoma from cholangiocarcinoma [14]. In non-keratinizing squamous epithelium, CK19 expression is normally confined to the basal layer, while it is typically absent in keratinizing squamous epithelium [15]. Its upregulation in dysplastic and malignant squamous lesions, such as those of the cervix and upper aerodigestive tract, has made it a valuable tool for detecting early neoplastic transformation [16,17].

Epstein–Barr virus (EBV) plays a central etiological role in non-keratinizing nasopharyngeal carcinoma (NPC), particularly in endemic regions such as Southeast Asia. Consequently, EBV serological assays—especially IgA antibodies against viral capsid antigen (VCA) and early antigen (EA)—have been incorporated into population-based screening protocols. Elevated EBV serology is associated with increased NPC risk and has demonstrated utility in early detection programs [18,19]. However, these tests exhibit variable sensitivity and specificity, as latent EBV infections are prevalent in the general population, and some early-stage or EBV-negative NPC subtypes may evade serologic detection [5,20]. Such limitations constrain the reliability of EBV serology as a standalone screening tool and underscore the need for more universally applicable biomarkers.

Cytokeratins (CKs), particularly CK8/18 and CK19, have demonstrated diagnostic value in various epithelial malignancies. While their expression in NPC has been explored via molecular techniques, there remains a paucity of immunohistochemical (IHC) data—especially within Malaysian populations, where the disease burden is significant. This diagnostic gap hinders the development of tissue-based screening adjuncts and may limit diagnostic precision in histologically ambiguous cases. Therefore, this study aims to evaluate the IHC expression patterns of CK8/18 and CK19 in NPC versus benign nasopharyngeal tissue to assess their potential as reliable markers for early diagnosis and screening in high-incidence regions.

## 2. Materials and Methods

### 2.1. Sample Size Calculation

Sample size was determined using an online calculator for comparing two proportions (Select Statistical Consultants Ltd., *Comparing Two Proportions—Sample Size Calculator*, https://select-statistics.co.uk/calculators/sample-size-calculator-two-proportions/) (accessed on 30 April 2025) employing the formula *n* = (Zα/2 + Zβ)^2^ × (p1(1 − p1) + p2(1 − p2))/(p1 − p2)^2^, with α = 0.05 and power = 80%. Confidence level was set at 95%.

Separate calculations were performed for both biomarkers, with the larger sample size selected. For CK19, based on expected proportions of 65% in NPC [21] versus 20% in benign tissue [15], 16 cases per group were required. For CK8/18, using oesophageal carcinoma as a surrogate for NPC, expected proportions were 40% versus 0% in benign tissue [12], requiring 12 cases per group. The sample size calculation was based on previously published data on CK8/18 expression in oesophageal squamous cell carcinoma, due to the absence of directly comparable data in nasopharyngeal carcinoma (NPC). This approach was selected because both tumours share squamous epithelial origins and exhibit overlapping immunophenotypic characteristics. While this surrogate model offers a practical estimation framework, we acknowledge that differences in tumour microenvironment, anatomical location, and molecular behaviour may limit the direct applicability of these findings to NPC.

The study adopted the larger requirement of 16 cases per group. Accounting for a 10% rate of inadequate tissue for immunohistochemical staining, the final sample size was set at 20 cases each of NPC and benign nasal tissue.

### 2.2. Tissue Specimens

A total of 62 cases labelled as nasal tissue or Fossa of Rosenmüller (FOR) were received by the Department of Pathology, Hospital Canselor Tuanku Muhriz, Kuala Lumpur between April 2021 and April 2024. Of these, 36 cases were diagnosed as malignant tumours based on WHO histopathological criteria, 23 were benign, and 3 had an indeterminate diagnosis. Eleven malignant cases were excluded due to diagnoses other than nasopharyngeal carcinoma, and one case was excluded due to exhaustion of diagnostic material. Among the benign group, one case was excluded due to extensive necrosis and the presence of fungal elements. The three indeterminate cases characterized by scant tissue with atypical cells were also excluded. The benign cases included in the study comprised diagnoses of non-specific benign nasal tissue (negative for malignancy, 11 cases), reactive lymphoid hyperplasia (8 cases), chronic inflammation (1 case), inflammatory polyp (1 case), and inverted papilloma (1 case). Histopathological slides of all cases were reviewed, with each tissue specimen considered as a single case. The corresponding formalin-fixed, paraffin-embedded (FFPE) tissue blocks were subsequently retrieved from the pathology archive for immunohistochemical analysis.

This study was conducted in accordance with the Declaration of Helsinki and approved by the UKM Research Ethics Committee, under protocol number UKM PPI/111/8/JEP-2023-459 and date of approval 20 July 2023. The need for informed consent was waived due to the retrospective and anonymized nature of the study.

### 2.3. Immunohistochemical Staining

Immunohistochemical staining was performed on tissue sections using the EnVision^TM^ FLEX Mini Kit, High pH (Code K8023, DAKO), following the manufacturer’s protocol. The primary antibodies utilized were FLEX monoclonal rabbit anti-human CK8/18 (Clone EP17/EP30) and FLEX monoclonal mouse anti-human CK19 (Clone RCK108), both provided in ready-to-use formulations by DAKO. For optimization, the primary antibodies were diluted to a working concentration of 1:100 using EnVision^TM^ FLEX Antibody Diluent (Code K8006, DAKO). All washing steps between reagent applications were carried out using EnVision^TM^ FLEX Wash Buffer (20×) (Code K8007, DAKO).

Two 4 µm-thick sections were prepared from the selected formalin-fixed, paraffin-embedded (FFPE) tissue blocks and mounted on FLEX IHC microscope slides (Product No. K8020-100, DAKO). The slides were subsequently deparaffinized and rehydrated through a series of graded alcohols. Heat-induced epitope retrieval (HIER) was performed by incubating the slides in pre-heated EnVision^TM^ FLEX Target Retrieval Solution.

Heat-induced epitope retrieval was performed using EnVision^TM^ FLEX Target Retrieval Solution, High pH (50×) (Code K8004, DAKO), by incubating the slides at 97 °C for 20 min. Following retrieval, one slide was incubated with FLEX monoclonal rabbit anti-human CK8/18 (Clone EP17/EP30), and the other with FLEX monoclonal mouse anti-human CK19 (Clone RCK108), according to the standardized protocol provided by the Autostainer Link software. After staining, the slides were dehydrated, mounted, and coverslipped for preservation. Tumour cells exhibiting antibody binding demonstrated a cytoplasmic staining pattern. Thyroid tissue was used as the positive control for CK8/18, while tonsillar and placental tissues served as positive controls for CK19. Normal nasopharyngeal tissue was used as the negative control.

### 2.4. Immunohistochemical Evaluation

All cases were independently reviewed by two pathologists to minimise observer bias. Immunoreactivity for CK8/18 and CK19 was interpreted semi-quantitatively using the categorical criteria adapted from Menz, Bauer, et al. [15] (Table 1), without computation of a composite immunoreactivity score. Cases were assigned to one of the four categories based on staining intensity and proportion of positive tumour cells: negative (no staining), weak (1 + intensity in ≤70% of cells or 2 + intensity in ≤30%), moderate (1 + in >70%, 2 + in 31–70%, or 3 + in ≤30%), and strong (2 + in >70% or 3 + in >30%). Intensity was judged on a 3-tier scale (1 + weak, 2 + moderate, 3 + strong) relative to internal controls. Proportion estimates were made visually in 10 high-power fields. No digital image analysis software (e.g., ImageJ) was used. Discrepancies were resolved at a multi-headed microscope to reach consensus. A *p*-value of <0.05 was considered statistically significant.

### 2.5. Statistical Analysis

Statistical analysis was performed using the *Statistical Package for the Social Sciences* (*SPSS*) for Windows, version 29.0. Categorical variables were compared between malignant and benign groups using the chi-square test for univariate analysis. A two-tailed *p*-value of <0.05 was considered statistically significant.

## 3. Results

### 3.1. Demographics and Clinicopathological Data

A total of 24 nasopharyngeal carcinoma (NPC) cases and 22 benign cases were evaluated for cytokeratin 8/18 (CK8/18) and cytokeratin 19 (CK19) immunohistochemical expression. The mean age at diagnosis was 51 years (range: 16–78 years) in the malignant group and 53 years (range: 23–74 years) in the benign group. Males predominated in the NPC group (66.6%), whereas the gender distribution was relatively equal in the benign group. Both groups demonstrated a similar ethnic distribution, with Malays comprising the majority (50% in each group), followed by Chinese, Indian, and other ethnicities (Table 2). All 24 NPC cases were of the non-keratinizing subtype. The benign group comprised cases with the following diagnoses: negative for malignancy (11 cases), reactive lymphoid hyperplasia (8 cases), chronic inflammation (1 case), inflammatory polyp (1 case), and inverted papilloma (1 case).

### 3.2. Comparison of Cytokeratin 8/18 and Cytokeratin 19 Expression in Nasopharyngeal Carcinoma and Benign Nasal Tissue

Upon stratification of the benign control group, CK19 and CK8/18 expression were absent in all cases of non-neoplastic tissue including reactive lymphoid hyperplasia and inflammatory conditions. Focal weak positivity was observed only in the case of inverted papilloma, which is a benign neoplasm suggesting that CK expression in benign tissue is not uniform and may be lesion-dependent. This further underscores the importance of contextual histopathological interpretation.

**Figure 1:**
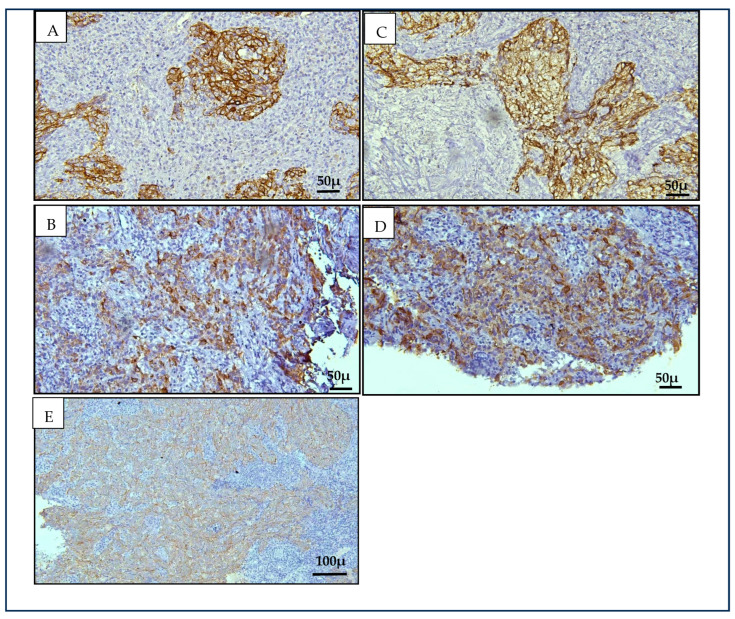
CK19 with strong (**A**) and moderate (**B**) expressions. CK8/18 strong (**C**), moderate (**D**), and weak (**E**) expression [Original magnifications as indicated; scale bars 100 µm (×200) and 50 µm (×400)].

Cytokeratin 8/18 and Cytokeratin 19 demonstrated significantly higher positivity in nasopharyngeal carcinoma (NPC) cases compared to benign controls (*p* < 0.001). Among the benign cases, only one (4.5%) showed positivity for both CK8/18 and CK19, exhibiting weak staining for CK8/18 and moderate staining for CK19 (Figure 1). The remaining 95.5% of benign samples were negative for both markers. In contrast, CK19 expression was consistently positive in all NPC cases (100%), with the majority demonstrating strong staining intensity (79.2%). CK8/18 expression in NPC was predominantly weak (50.0%), while one case (4.2%) showed no detectable staining. Based on this data, CK19 showed a sensitivity of 85.75% to 100% (CI = 95%) for NPC detection, outperforming CK8/18, which had a sensitivity of 78.88 to 99.89% (CI = 95%). The specificity for both CK19 and CK8/18 was comparable at 77.16% to 99.88% (CI = 95%) (Table 3).

### 3.3. Cytokeratin 8/18 and Cytokeratin 19 Expression in Nasopharyngeal Cases with Positive EBV Status

Epstein–Barr encoding region (EBER) in situ hybridization was performed on 15 of the 24 NPC cases, all of which were EBER-positive. Among these, CK19 expression was consistently present (100%), with strong staining observed in 12 cases (80%) and moderate staining in the remaining 3 cases (20%). CK8/18 expression was more variable: only 2 cases (13.3%) demonstrated strong positivity, 12 cases (80%) showed weak to moderate staining, and 1 case (6.7%) was negative (Table 4). These findings reinforce the consistent expression of CK19 in EBV-associated NPC, while CK8/18 appears more heterogeneously expressed.

## 4. Discussion

A total of 24 cases of nasopharyngeal carcinoma (NPC) were diagnosed at Hospital Canselor Tuanku Muhriz, Universiti Kebangsaan Malaysia, between April 2021 and April 2024. The patients’ ages ranged from 16 to 78 years, with a mean age of 51.0 ± 14.74 years, consistent with findings from local studies, such as that by Azmir et al. [22]. The male-to-female ratio in our cohort was 2:1, which is slightly lower than the national average of 2.8:1, though male predominance remains evident. Notably, 50% of the NPC cases in our cohort were among Malay patients, which differs from the national trend of higher NPC incidence among individuals of Chinese ethnicity. This variation likely reflects the demographic profile of patients presenting to our institution rather than a shift in underlying disease epidemiology. All NPC cases in this study were of the non-keratinizing subtype (NK-NPC), which aligns with the known predominance of this histological subtype. Consequently, the findings of this study may not be directly applicable to other subtypes, such as keratinizing (K-NPC) or basaloid (B-NPC) NPC. Henceforth, references to NPC in this discussion pertain specifically to the NK-NPC subtype. Therefore, the diagnostic utility of CK8/18 and CK19 as described in this study should be interpreted within the context of NK-NPC only. The expression profiles observed may not be representative of keratinizing or other rarer histological variants of NPC, which require separate investigation.

Nasopharyngeal carcinoma (NPC) is a malignant epithelial tumour of the nasopharyngeal mucosa that typically exhibits squamous differentiation. The non-keratinizing subtype, which predominates in endemic regions, is characterized by poorly differentiated epithelial cells with minimal keratinization and a variably dense lymphoid stroma. In challenging biopsies, immunohistochemistry (IHC) for pan-cytokeratin and Epstein–Barr virus-encoded RNA (EBER) in situ hybridization (ISH) are recommended. In this study, 37.5% of NPC cases were diagnosed based on morphology alone, while the remaining 62.5% were confirmed using ancillary studies, including combinations of CKAE1/AE3 with EBER ISH (13%) and CK5/6 with EBER ISH (13%). Additional markers such as CD3, CD20, and LCA were used to exclude lymphoid malignancies and were negative.

Our findings revealed significant immunopositivity for Cytokeratin 8/18 (CK8/18) and Cytokeratin 19 (CK19) in NK-NPC cases compared to benign controls (*p* < 0.001), supporting our hypothesis based on data from squamous cell carcinomas at other anatomical sites [15]. Both markers displayed a cytoplasmic staining pattern. CK19 showed stronger and more consistent staining than CK8/18, with 79.2% of NPC cases demonstrating strong CK19 expression. In contrast, CK8/18 showed predominantly weak positivity (50%), with one case (4.2%) negative for expression. All NK- NPC cases in this study were positive for CK19, contributing to its higher sensitivity (100%) compared to CK8/18 (92%). The specificity for both markers was equivalent at 95.5%. When combined, there was only a marginal increase in specificity (from 95.5% to 96%). The diagnostic relevance of CK8/18 and CK19 in head and neck malignancies has been previously reported, with increased expression noted in premalignant and malignant epithelial transformations [23,24]. CK19, in particular, has demonstrated high sensitivity in large tissue-based analyses across multiple tumour types, supporting its potential diagnostic value [15]. The consistent overexpression of CK19 in all NK-NPC cases evaluated in this study highlights its potential as an adjunct immunohistochemical marker to support histopathological diagnosis, particularly in diagnostically ambiguous cases.

Stratification of benign controls confirmed that CK8/18 and CK19 expression was limited to the inverted papilloma case, while all other benign lesions—including reactive hyperplasia and cysts—were negative. This finding highlights the specificity of these markers in distinguishing NPC from common non-neoplastic nasopharyngeal tissues, though occasional overlap with benign epithelial proliferations should be interpreted with caution.

Given the well-recognized limitations of EBV serology, particularly its reduced specificity due to widespread latent infection, adjunct tissue-based markers remain important for diagnostic confirmation at the histopathological level. In our study, CK19 was consistently expressed in all EBER-positive NK-NPC cases, supporting its potential as a complementary marker in routine diagnostic workup. While EBV serology and EBER-ISH remain central to NPC diagnosis and surveillance, CK19 immunohistochemistry may provide additional value in morphologically ambiguous biopsies, particularly when used alongside pan-cytokeratin panels.

Our EBER sub-analysis highlights the diagnostic potential of CK19 in EBV-associated NK-NPC. All EBER-positive cases (*n* = 15) expressed CK19, with 80% showing strong intensity staining. This consistency suggests CK19 may serve as a reliable tissue marker in EBV-driven NK-NPC, particularly in non-keratinizing subtypes where histological features may overlap with benign conditions. In contrast, CK8/18 showed variable expression among EBER-positive cases, with only two cases exhibiting strong staining and one case completely negative.

It is important to distinguish the dual relevance of the study by Menz et al. [15], which has been cited in this paper both as the basis for the semi-quantitative immunohistochemistry (IHC) scoring system and as supporting evidence for CK19 expression in epithelial malignancies. While the scoring criteria used in our study were adapted from Menz et al.’s methodological framework—specifically their detailed intensity-based scoring applied across a broad tumour cohort—the same study also contributed significant evidence regarding CK19 expression patterns across diverse tumour types. To avoid conflating methodological justification with validation of findings, we emphasize that our use of their scoring system pertains strictly to its structured approach to IHC quantification, while the biological insights drawn from their tumour profiling data serve as independent support for the diagnostic relevance of CK19. These two distinct applications reinforce, but should not be interpreted as circular validation.

The benign cohort in this study included cases diagnosed as negative for malignancy (11 cases), reactive lymphoid hyperplasia (8 cases), chronic inflammation (1 case), inflammatory polyp (1 case), and inverted papilloma (1 case). Histologically, the nasopharynx consists predominantly of lymphoid stroma lined by respiratory-type epithelium, especially at the fossa of Rosenmüller—an anatomic region frequently involved in NPC. In these tissues, cytokeratin stains are typically negative in lymphoid cells but positive in scattered seromucinous glands and the respiratory epithelium.

In tissues from other parts of the nasal cavity, differences in epithelial lining influence cytokeratin staining. The stratified squamous epithelium (e.g., from the vestibular region) is typically negative for CK8/18 and CK19, while respiratory-type epithelia exhibit positive staining. These findings align with previous observations that normal squamous cells are negative for CK8/18 and CK19, whereas the glandular epithelium and malignant squamous cells show positivity. This highlights the importance of correlating immunostaining results with morphological features.

To our knowledge, this is the first study to comprehensively evaluate the immunohistochemical expression of CK8/18 and CK19 in nasopharyngeal carcinoma using a semi-quantitative scoring system within a Malaysian cohort. Prior studies have explored the molecular profiles of cytokeratins in NPC, on the other hand our findings provide direct tissue-level evidence supporting their diagnostic relevance, particularly in distinguishing malignant from benign nasopharyngeal lesions. This work contributes novel regional data and reinforces the potential of CK19 as a high-sensitivity adjunct marker, thereby addressing a critical gap in the existing diagnostic landscape for NPC in endemic populations. The diagnosis of non-keratinizing nasopharyngeal carcinoma (NK-NPC) is primarily based on histological features and confirmation with EBER in situ hybridization. However, in limited biopsies, or those with intense inflammation, additional immunohistochemical (IHC) markers such as pan-cytokeratin (CKAE1/AE3), CK5/6, and LMP1 are often employed to aid in distinguishing NK-NPC from benign lymphoid proliferations, lymphomas, or poorly differentiated non-nasopharyngeal carcinomas. Although CK8/18 and CK19 are not routinely used in the diagnostic workup of NPC, their consistent expression in our cohort suggests potential complementary value in such diagnostically challenging scenarios. We acknowledge that our study did not specifically assess equivocal cases, and thus, further investigation is needed to determine the utility of CK8/18 and CK19 in these contexts.

## 5. Limitations and Future Directions

This study has several limitations that should be acknowledged. Firstly, the sample size was relatively small and limited to a single tertiary referral centre, which may reduce the generalizability of the findings to broader populations. Additionally, only non-keratinizing nasopharyngeal carcinoma (NK-NPC) cases were included, and thus, the utility of CK8/18 and CK19 immunohistochemistry in other NPC subtypes, such as keratinizing and basaloid variants, remains unassessed. Another limitation is the lack of comparison with other benign epithelial neoplasms or non-NPC malignancies, which may present diagnostic challenges in real-world practice. Furthermore, EBER in situ hybridization, while used in some cases, was not uniformly applied across all NPC cases, limiting assessment of its correlation with cytokeratin expression. Another limitation of this study is the use of oesophageal squamous cell carcinoma data as a surrogate for NPC in the sample size estimation. Although histologically related, these tumours arise from distinct anatomical and molecular contexts, which may affect cytokeratin expression profiles.

Future studies should aim to validate these findings in larger, multi-centre cohorts and include a wider spectrum of nasopharyngeal lesions. Additional exploration into the diagnostic performance of CK8/18 and CK19 in small, limited biopsies, as well as their potential prognostic or therapeutic implications in NPC, may further enhance their clinical utility.

## 6. Conclusions

This study demonstrates that CK8/18 and CK19 are significantly overexpressed in non-keratinising nasopharyngeal carcinoma (NK-NPC) compared to benign nasopharyngeal tissues, supporting their utility as adjunct immunohistochemical markers in diagnostic evaluation. CK19 exhibited strong and consistent expression, yielding high sensitivity and specificity, making it a promising candidate for histopathological differentiation of NPC from benign lesions. CK8/18, while less robust in staining intensity, contributed additional diagnostic value when interpreted in conjunction with CK19. Both markers can also be expressed in benign glandular structures, and their strong and widespread positivity in malignant tissues supports their potential diagnostic relevance when interpreted in appropriate clinical and morphological contexts. While these results are encouraging, the findings are preliminary and should be interpreted cautiously. Further studies with larger, multi-centre cohorts are required to validate these markers’ diagnostic performance and to define their role within broader IHC panels for NPC diagnosis.

It is important to note that these findings are specific to the non-keratinizing subtype and should not be generalized to other histological variants of NPC without further validation. While other studies have reported associations between cytokeratin expression and tumour aggressiveness, our findings are diagnostic in nature and do not address prognostic correlations. Further investigation in larger, outcome-linked cohorts would be required to explore prognostic implications.

Further prospective studies are needed to assess whether CK19 and CK8/18, in combination with EBV-based assays, can enhance diagnostic accuracy in routine clinical workflows. Such investigations could also help clarify their utility in high-prevalence or resource-limited settings. Our findings provide an initial basis for considering these cytokeratins in diagnostic panels, but any integration into standardized diagnostic or screening protocols will require broader validation, including assessment of feasibility, reproducibility, and clinical impact.

## Figures and Tables

**Table 1:** CK8/18 and CK19 immunohistochemistry scoring system [15].

IHC Grading	Description
Negative	No staining
Weak	1 + staining intensity in ≤70% of cells or 2 + intensity in ≤30% of cells
Moderate	1 + staining intensity in >70% of cells, 2 + intensity in 31 to 70% of cells, or 3 + intensity in ≤30% of cells
Strong	2 + intensity in >70% or 3 + intensity in >30% of cells

**Table 2:** Demographic data along with CK8/18 and CK19 expression in NPC and benign cases.

Variable	Nasopharyngeal Carcinoma (*n* = 24)	Benign Cases (*n* = 22)	Total (*n* = 46)
Mean Age (years)	51 (SD 14.74)	53 (SD 15.32)	
Gender			
Female	8 (33.3%)	12 (54.5%)	20 (43.5%)
Male	16 (66.6%)	10 (45.5%)	26 (56.5%)
Ethnicity			
Malay	12 (50.0%)	11 (50.0%)	23 (50.0%)
Chinese	9 (37.5%)	9 (40.9%)	18 (39.1%)
Indian	0 (0.0%)	1 (4.5%)	1 (2.2%)
Foreigner	3 (12.5%)	1 (4.5%)	4 (8.7%)
CK8/18 Expression			
Negative	1 (4.2%)	21 (95.5%)	22 (47.8%)
Weak	12 (50.0%)	1(4.5%)	22 (47.8%)
Moderate	8 (33.3%)	0 (0%)	8 (17.4%)
Strong	3 (12.5%)	0 (0%)	3 (6.5%)
CK19 Expression			
Negative	0 (0%)	21 (95.5%)	21 (45.7%)
Weak	1 (4.2%)	0 (0%)	1 (2.2%)
Moderate	4 (16.7%)	1 (4.5%)	5 (10.9%)
Strong	19 (79.2%)	0 (0%)	19 (41.3%)

**Table 3:** Comparison of CK8/18 and CK19 expression in benign cases according to diagnosis.

Diagnosis	Total Case	CK8/18 Positive	CK19 Positive
Negative for malignancy	11	0 (0%)	0 (0%)
Reactive lymphoid hyperplasia	8	0 (0%)	0 (0%)
Inflammatory conditions	3	0 (0%)	0 (0%)
Inverted papilloma	1	1 (100%)	1 (100%)
All Benign Cases	22	1 (4.5%)	1 (4.5%)

**Table 4:** C8/18 and CK19 expression in EBER-ISH positive cases.

Staining Intensity	CK8/18 (*n* = 15)	CK19 (*n* = 15)
Negative	1 (6.7%)	0 (0%)
Weak	6 (40%)	1 (6.7%)
Moderate	6 (40%)	2 (13.3%)
Strong	2 (13.3%)	12 (80%)

## Data Availability

The data presented in this study is contained within the article material.
